# Physical activity, mental-health symptoms, sleep quality, and health-related quality of life among physical education teacher education students: a cross-sectional study

**DOI:** 10.3389/fpubh.2026.1833793

**Published:** 2026-07-09

**Authors:** Eugenio Merellano-Navarro, Paula Loyola-Arriagada, Antonia Moran-Toloza, José Bruneau-Chávez, Frano Giakoni-Ramírez, Dilan Gajardo-Herrera, Joaquín Pena-Rojas, Andrés Godoy-Cumillaf

**Affiliations:** 1 Department of Physical Activity Sciences, Faculty of Education Sciences, Universidad Católica del Maule, Talca, Chile; 2 Departamento de Educación Física, Deportes y Recreación, Universidad de la Frontera, Temuco, Chile; 3 Faculty of Education and Social Sciences, Universidad Andres Bello, Las Condes, Santiago, Chile; 4 Grupo de Investigación en Educación Física, Salud y Calidad de Vida (EFISAL), Facultad de Educación, Universidad Autónoma de Chile, Temuco, Chile

**Keywords:** depression, health-related quality of life, physical activity, sleep quality, teacher education, university students

## Abstract

**Background:**

Health-related quality of life (HRQoL) among university students is associated with behavioral, psychological, and lifestyle factors. Although physical activity is widely recognized as beneficial for health, the associations of mental health symptoms and sleep quality with HRQoL remain insufficiently explored, particularly among physical education teacher education students. This study aimed to examine the associations between physical activity, mental health symptoms, sleep quality, and HRQoL among physical education teacher education students, accounting for sex and academic cohort.

**Methods:**

A cross-sectional study was conducted among 168 university students in Chile. Physical activity was assessed using the Global Physical Activity Questionnaire, mental health symptoms with the Depression, Anxiety and Stress Scale-21, sleep quality with the Pittsburgh Sleep Quality Index, and HRQoL with the 12-Item Short Form Health Survey version 2. Multivariable linear regression models were used to examine associations with the physical component summary (PCS) and mental component summary (MCS) scores.

**Results:**

Participants reported high levels of physical activity and a mean PSQI score of 9.71 ± 2.77, indicating poor overall sleep quality according to established PSQI criteria. Depressive symptoms were negatively associated with both PCS and MCS, with stronger associations observed for MCS. Physical activity was positively associated with MCS, but not with PCS. The model explained substantially more variance in MCS than in PCS (*R*^2^ = 0.61 vs. *R*^2^ = 0.06). Academic cohort was also significantly associated with MCS in the adjusted model.

**Conclusion:**

In this sample of physically active teacher education students, HRQoL showed stronger associations with mental health indicators than with the physical activity measure included in the models.These findings should

be interpreted in the context of a young and physically active sample, in which variability in physical activity and perceived physical health may have been limited. Given the cross-sectional design, the observed associations should not be interpreted as evidence of longitudinal change or causal relationships.

## 1 Introduction

Non-communicable diseases (NCDs) are among the leading causes of morbidity and mortality worldwide and are strongly associated with behavioral and psychosocial risk factors that tend to consolidate during early adulthood (1). Universities represent critical environments for health promotion during this life stage, as the transition to higher education is frequently accompanied by substantial lifestyle changes, academic overload, and increased exposure to psychosocial stressors. These changes may negatively affect mental health, sleep quality, and health-related quality of life (HRQoL) among university students (2–5).

Mental health problems among university students have become a growing global public health concern. Large international studies have reported high prevalence rates of depression, anxiety, and psychological distress within university populations, conditions that may significantly affect both academic performance and overall wellbeing (6). Systematic reviews have further highlighted the high prevalence of depressive symptoms among university students worldwide, reinforcing the relevance of mental health as a key public health issue in higher education contexts (7). Large international studies have reported high prevalence rates of psychological distress among university students (8).

Lifestyle behaviors such as physical activity and sleep patterns are important factors associated with health and wellbeing during early adulthood. Regular physical activity has been consistently associated with multiple physical and psychological health benefits, including reductions in depressive symptoms and improvements in perceived quality of life (9). Similarly, adequate sleep duration and good sleep quality play an essential role in maintaining cognitive functioning, emotional regulation, and overall wellbeing (10–12). These lifestyle behaviors interact with psychological factors, and are related to the overall perception of health and wellbeing among young adults. Physical activity and sleep quality were included in the present study as key lifestyle behaviors consistently associated with mental health and wellbeing in university populations.

Health-related quality of life (HRQoL) is a multidimensional construct encompassing physical, psychological, and social aspects of wellbeing (13, 14). It has been widely used in public health research as a comprehensive indicator to assess how behavioral and psychosocial factors are associated with population health. Understanding the correlates of HRQoL among university students is particularly relevant, as health behaviors and wellbeing patterns developed during this life stage may persist into adulthood and be associated with long-term health trajectories (15).

From a public health perspective, identifying the behavioral and psychosocial factors associated with HRQoL among university students is essential for designing effective health promotion strategies within educational environments. Although physical activity has traditionally been considered an important correlate of wellbeing, accumulating evidence suggests that psychological factors, particularly depressive symptoms, may show equally strong or even stronger associations with perceived wellbeing among young adults (16–18). In this sense, understanding the relative contribution of lifestyle behaviors and mental health indicators becomes especially relevant for explaining variations in HRQoL among university students. The variables included in this study were selected within a behavioral and psychosocial framework and are conceptualized as correlates of HRQoL rather than causal determinants, given the cross-sectional design.

This issue becomes particularly relevant among students enrolled in physical education teacher education programs. Due to the practical and movement-based nature of their academic training, these students often report higher levels of physical activity than the general university population (17, 19). Nevertheless, previous research suggests that even in physically active populations, significant levels of academic stress, depressive symptoms, and reduced wellbeing may still occur (16, 17).

Although lifestyle behaviors have been widely examined in university populations, physically active groups such as physical education teacher education students have received comparatively less attention. In these contexts, the relatively low variability in physical activity levels provides a unique opportunity to examine the relative contribution of behavioral and psychological factors to health-related quality of life. Because of the practical and movement-based nature of their academic training, these students often report higher levels of physical activity than the general university population (17, 19). Although previous studies have examined the relationships among physical activity, mental health, sleep quality, and HRQoL in university students, evidence remains limited in physical education teacher education students, particularly in Latin American contexts and when considering the physical and mental components of HRQoL separately. Therefore, the present study aimed to examine these associations among Chilean physical education teacher education students, providing a complementary perspective to findings reported in general university populations. Therefore, the aim of the present study was to examine the associations between physical activity, mental health symptoms, sleep quality, and health-related quality of life among physical education teacher education students, accounting for sex and academic cohort. Specifically, this study examined the associations of these factors with the physical and mental components of HRQoL in this population.

## 2 Methods and materials

### 2.1 Study design

An observational, analytical, cross-sectional study was conducted to examine the associations between physical activity, mental health symptoms, sleep quality, and health-related quality of life (HRQoL) among students enrolled in Physical Education teacher education programs. The cross-sectional design allowed the assessment of how behavioral and psychological variables were associated with the physical and mental components of HRQoL.

### 2.2 Participants and sampling

The study population consisted of 294 students enrolled in the Physical Education teacher education program at a Chilean university. A non-probabilistic convenience sampling approach was used, inviting students from the active academic cohorts entering between 2022 and 2025 to participate.

The minimum sample size was calculated assuming a finite population, a 95% confidence level, a 5% margin of error, and an expected proportion of 0.5, resulting in a required minimum sample of 167 students. To account for potential non-response, the entire eligible population was invited to participate. The final analytical sample consisted of 168 students who met the inclusion criteria, did not meet any exclusion criteria, and provided complete data for all study variables. Participants with missing information in one or more study instruments were excluded from the analyses. Consequently, all statistical analyses were conducted using complete-case data. Additionally, the sample size was considered in relation to the planned multivariable regression analyses. Following general recommendations for linear regression models, which suggest an adequate ratio of observations per predictor variable, the available sample was deemed sufficient for the number of predictors included in the models, although this also limited the inclusion of additional covariates. Participants were recruited through non-probabilistic convenience sampling from a single university; therefore, the findings should not be considered representative of all physical education teacher education students.

The inclusion criteria were: (i) being 18 years of age or older, (ii) being enrolled in one of the active cohorts of the program, and (iii) voluntarily agreeing to participate by providing written informed consent. Questionnaires with incomplete responses or inconsistent information were excluded from the analysis.

### 2.3 Procedures

Data were collected in person between August and October 2025 at university facilities, primarily in classroom settings. Prior to data collection, participants received standardized information regarding the objectives of the study, the voluntary nature of participation, and the confidentiality of the data. The estimated time required to complete the questionnaires was approximately 20 min. The study was approved by the institutional Ethics Committee (CEC 26–24) and was conducted in accordance with the principles of the Declaration of Helsinki.

### 2.4 Variables and instruments

#### 2.4.1 Health-related quality of life (HRQoL)

HRQoL was assessed using the Spanish version of the 12-Item Short Form Health Survey version 2 (SF-12 v2). The SF-12 comprises 12 items covering eight health domains: physical functioning, role physical, bodily pain, general health, vitality, social functioning, role emotional, and mental health. Responses were scored according to the standard SF-12 v2 scoring procedures, generating two summary measures: the Physical Component Summary (PCS) and the Mental Component Summary (MCS). Summary scores were transformed to a 0–100 scale, with higher scores indicating better perceived health-related quality of life. The SF-12 v2 has demonstrated adequate validity and reliability across diverse adult populations and has been widely used in epidemiological and public health research (20).

#### 2.4.2 Physical activity

Physical activity was assessed using the Spanish version of the Global Physical Activity Questionnaire (GPAQ v2), developed by the World Health Organization. The instrument evaluates physical activity performed during a typical week across three domains: work/study activities, transportation, and leisure-time physical activity, distinguishing between moderate- and vigorous-intensity activities (21). The GPAQ has demonstrated acceptable reliability and validity across diverse international populations, including university-aged adults (22). For the present study, total physical activity was calculated as the sum of minutes per week accumulated across all domains. Data were reviewed according to WHO GPAQ recommendations to identify inconsistent or implausible responses. Total physical activity (minutes/week) was used as a continuous variable in all analyses, and responses containing implausible values were excluded.

#### 2.4.3 Mental health

Symptoms of depression, anxiety, and stress were assessed using the validated Spanish version of the Depression, Anxiety and Stress Scale-21 (DASS-21), which has demonstrated adequate psychometric properties in Chilean university students (23). The instrument consists of 21 items grouped into three subscales: Depression, Anxiety, and Stress, each comprising seven items. Responses are rated on a four-point Likert scale ranging from 0 (“Did not apply to me at all”) to 3 (“Applied to me very much or most of the time”). Subscale scores were calculated by summing the corresponding items and multiplying the total by two, according to standard DASS-21 scoring procedures, producing scores comparable to those of the original 42-item version. Higher scores indicate greater symptom severity. Continuous scores were used in all analyses (24).

#### 2.4.4 Sleep quality

Sleep quality was assessed using the Spanish version of the Pittsburgh Sleep Quality Index (PSQI). The PSQI evaluates subjective sleep quality during the previous month through 19 self-reported items that generate seven component scores: subjective sleep quality, sleep latency, sleep duration, habitual sleep efficiency, sleep disturbances, use of sleep medication, and daytime dysfunction. These components are summed to obtain a global score ranging from 0 to 21, with higher scores indicating poorer sleep quality. A global score >5 is commonly interpreted as indicative of poor sleep quality. In the present study, the PSQI global score was analyzed as a continuous variable (25).

All instruments corresponded to previously validated Spanish-language versions with documented evidence of validity and reliability in Spanish-speaking populations. Reliability coefficients from the original validation studies are reported in the respective validation articles cited above.

Covariates: analyses were adjusted for sex (0 = women, 1 = men) and academic cohort of entry. Academic cohort was entered as an ordinal variable representing progression through the academic trajectory (from first- to fourth-year students). Academic cohort was used as a proxy for progression in the teacher education program, although it may not fully capture variability in academic demands or individual experiences. These variables were included as covariates based on prior evidence showing their association with physical activity, mental health, and health-related quality of life in university populations.

### 2.5 Statistical analysis

The normality of the distributions was assessed using the Kolmogorov–Smirnov test, complemented by skewness and kurtosis indices and visual inspection of histograms. Differences between men and women were examined using independent samples Student's *t*-tests (Table 1), whereas comparisons according to academic cohort were performed using one-way analysis of variance (ANOVA) (Table 2). The magnitude of group differences was estimated using effect sizes, calculated as Cohen's *d* for *t*-tests and partial eta squared (η^2^*p*) for ANOVA. Bivariate associations between physical activity, mental health symptoms, sleep quality, and health-related quality of life were examined using Pearson correlation coefficients (*r*) (Table 3). Subsequently, multivariable linear regression models were estimated to examine the independent associations between the variables of interest (Table 4). Two separate models were constructed using the PCS and MCS of the SF-12 v2 as dependent variables. The predictors included total physical activity (minutes per week), depression, anxiety, and stress scores (DASS-21), and sleep quality (PSQI), adjusting for sex and academic cohort. Results were reported as unstandardized coefficients (*B*), standard errors (SE), standardized coefficients (β), and 95% confidence intervals (95% CI), as well as *R*^2^.

**Table 1:** Differences in physical activity, mental health, sleep quality, and health-related quality of life according to sex.

Variable	Woman (*n* = 67) Mean ±SD	Men (*n* = 101) Mean ±SD	*p*	Cohen's *d*
Physical activity (min/week)	902 ± 718	1,222 ± 1,045	0.03	−0.34
Depression (DASS-21)	5.91 ± 4.86	4.64 ± 4.11	0.07	0.28
Anxiety (DASS-21)	6.10 ± 4.68	3.81 ± 3.19	< 0.001	0.59
Stress (DASS-21)	8.70 ± 4.84	7.16 ± 4.64	0.01	0.32
Sleep quality (PSQI)	10.31 ± 2.62	9.30 ± 2.79	0.01	0.38
Physical component summary (PCS)	52.23 ± 6.38	51.28 ± 6.83	0.38	0.14
Mental component summary (MCS)	44.68 ± 10.13	50.05 ± 9.88	0.01	−0.53

Effect sizes (Cohen's d) are reported to facilitate interpretation of group differences.

**Table 2:** Differences in physical activity, mental health, sleep quality, and health-related quality of life according to academic cohort.

Variable	Academic cohort				*p*	*n* ^2^ *p*
	2025 (*n* = 37)	2024 (*n* = 53)	2023 (*n* = 42)	2022 (*n* = 36)		
Physical activity (min/week)	1,110 ± 914	1,019 ± 730	983 ± 674	1,320 ± 1,396	0.03	0.01
Depression (DASS-21)	4.76 ± 5.05	4.34 ± 3.41	5.62 ± 4.16	6.19 ± 5.33	0.21	0.02
Anxiety (DASS-21)	5.08 ± 4.09	3.96 ± 3.81	5.43 ± 4.13	4.67 ± 4.02	0.32	0.02
Stress (DASS-21)	7.70 ± 4.53	6.68 ± 4.43	8.17 ± 4.82	9.00 ± 5.25	0.69	0.03
Sleep quality (PSQI)	8.62 ± 2.74	9.98 ± 2.97	9.48 ± 2.18	10.72 ± 2.76	< 0.001	0.06
Physical component summary (PCS)	50.01 ± 9.74	50.38 ± 8.71	45.01 ± 10.41	45.44 ± 11.69	0.06	0.04
Mental component summary (MCS)	52.05 ± 7.45	49.61 ± 6.51	52.47 ± 5.67	53.17 ± 6.59	0.02	0.06

Significant differences were observed across academic cohorts in physical activity, sleep quality, and the MCS. However, MCS values did not follow a clear monotonic pattern across cohorts, and cohort-related differences should therefore be interpreted cautiously. The effect size was small to moderate (η^2^*p* ≈ 0.06).

**Table 3:** Bivariate correlations between physical activity, mental health, sleep quality, and health-related quality of life.

Variable		PA	Depression	Anxiety	Stress	PSQI	PCS	MCS	Academic cohort
PA	*r*	—							
Depression	*r*	−0.09	—						
Anxiety	*r*	−0.07	0.68^***^	—					
Stress	*r*	−0.06	0.71^***^	0.67^***^	—				
PSQI	*r*	−0.07	0.45^***^	0.34^***^	0.49^***^	—			
PCS	*r*	−0.01	−0.19^*^	0.09	0.13	0.02	—		
MCS	*r*	0.27^***^	−0.70^***^	−0.56^***^	−0.63^***^	−0.44^***^	−0.37^***^	—	
Academic cohort	*r*	0.066	0.139	0.017	0.127	0.210^**^	0.109	−0.207^**^	—

Depressive symptoms showed a strong negative association with the MCS (*r* = −0.70, *p* < 0.001) and a moderate association with the Physical Component Summary (PCS) (*r* = −0.19, *p* < 0.05). Physical activity was positively associated with the MCS (*r* = 0.27, *p* < 0.001), but not with the PCS. The magnitude of the association between depressive symptoms and the MCS was more than twice that observed between physical activity and the MCS, suggesting a stronger association of depressive symptoms with perceived mental well-being in this sample. In addition, academic cohort was positively associated with poorer sleep quality and negatively associated with the MCS. The symbols indicate levels of statistical significance as follows: ^*^p < 0.05, ^**^p < 0.01, ^***^p < 0.001.

**Table 4:** Multivariable linear regression models for health-related quality of life.

Predictor	PCS				MCS			
	PCS B (SE)	95% CI	β	*p*	MCS B (SE)	95% CI	β	*p*
Physical activity (min/week)	0.0001 (0.0004)	−0.00 to 0.00	0.01	0.893	0.00 (0.001)	0.00 to 0.00	0.19	< 0.001
Depression (DASS-21)	−0.37 (0.18)	−0.74 to −0.01	−0.25	0.045	−1.00 (0.18)	−1.36 to −0.64	−0.44	< 0.001
Anxiety (DASS-21)	−0.17 (0.20)	−0.56 to 0.22	−0.10	0.385	−0.19 (0.20)	−0.58 to 0.20	−0.08	0.334
Stress (DASS-21)	−0.08 (0.17)	−0.41 to 0.25	0.06	0.649	−0.38 (0.17)	−0.73 to −0.04	−0.18	0.028
Sleep quality (PSQI)	−0.26 (0.22)	−0.70 to 0.18	−0.11	0.237	−0.29 (0.22)	−0.73 to 0.15	−0.08	0.197
Sex (men vs. women)	−1.17 (1.14)	−3.41 to 1.08	−0.09	0.306	2.41 (1.14)	0.17 to 4.66	0.12	0.035
Academic cohort	0.60 (0.51)	−0.41 to 1.60	0.10	0.242	−1.18 (0.51)	−2.18 to −0.18	−0.12	0.021
	Model fit: *R*^2^ = 0.06				Model fit: *R*^2^ = 0.61			

Prior to model estimation, linearity, normality of residuals, homoscedasticity, independence of errors (Durbin–Watson), multicollinearity (VIF < 5), and influential observations (Cook's distance) were assessed. Diagnostic analyses indicated no substantial violations of model assumptions. Given the observational and cross-sectional nature of the study, the regression analyses were interpreted as exploratory analyses of associations rather than confirmatory models. All statistical analyses were performed using IBM SPSS^®^ Statistics version 24, adopting a statistical significance level of p < 0.05 (two-tailed tests).

## 4 Results

Table 1 shows significant differences by sex in physical activity, anxiety, stress, sleep quality, and the MCS. Men reported higher levels of physical activity and better mental well-being, whereas women showed higher levels of anxiety and stress, as well as poorer sleep quality. Sex differences were observed primarily in the psychological dimension of well-being, as no significant differences were found in the PCS. Effect sizes were moderate for anxiety (*d* = 0.59) and MCS (*d* = −0.53).

In the adjusted model for the PCS, only depressive symptoms were negatively associated with the physical component (β = −0.25; *p* = 0.045). The model explained 6% of the variance (*R*^2^ = 0.06), indicating that the variables included had a limited contribution to physical health perception in this young and physically active population. Notably, physical activity was not independently associated with PCS after adjusting for psychological factors. In contrast, the model for the mental component (MCS) showed a substantially higher explanatory capacity (*R*^2^ = 0.61). Depressive symptoms (β = −0.44; *p* < 0.001) showed the strongest association, followed by physical activity (β = 0.19; *p* < 0.001), stress (β = −0.18; *p* = 0.028), sex, and academic cohort.

## 5 Discussion

The present study examined the associations between physical activity, mental health symptoms, sleep quality, and health-related quality of life (HRQoL) among students enrolled in Physical Education teacher education programs, considering differences by sex and academic cohort. Overall, the findings indicate that mental health indicators, particularly depressive symptoms, showed stronger associations with HRQoL than the physical activity measure included in the models. Depressive symptoms were consistently associated with both the physical and mental components of HRQoL, whereas physical activity was associated only with the mental component. Additionally, academic progression showed associations with psychological wellbeing, suggesting a potential decline in mental wellbeing as students advance through their academic trajectory. These findings highlight the relevance of psychosocial factors for understanding perceived wellbeing in this specific group of university students (26). These findings should be interpreted as associations between variables rather than causal effects, given the cross-sectional design of the study.

Notably, a marked asymmetry was observed in the explanatory capacity of the models, as the variables examined accounted for 61% of the variance in the mental component (MCS) but only 6% in the physical component (PCS). This difference suggests that the variables included in this study are considerably more strongly associated with the psychological dimension of HRQoL than with perceived physical health in this young and physically active population. This pattern should be interpreted in light of the characteristics of the sample, which was composed of students with generally high levels of physical activity and possibly limited variability in perceived physical health. This pattern is consistent with previous evidence reported in university student populations, although the present findings should be interpreted within the specific context of physically active teacher education students, reflecting the greater sensitivity of the MCS to emotional and psychological distress-related variables (27).

One of the most notable findings is the strong association of depressive symptoms with HRQoL. In the adjusted models, depressive symptoms were negatively associated with both components of the SF-12, with a particularly strong association observed for the mental dimension (β = −0.44). This finding is consistent with previous evidence identifying depression as an important factor associated with perceived wellbeing in university populations (28, 29), even in contexts where levels of physical activity are relatively high (30). In university settings, depressive symptoms have been associated not only with emotional distress but also with reduced academic functioning, lower social engagement, and poorer overall life satisfaction, reinforcing their close association with perceived health and wellbeing. The magnitude of the association between depressive symptoms and the MCS was substantially greater than that observed for physical activity (β = 0.19), indicating that, in this sample, depressive symptoms were more closely associated with perceived mental wellbeing than physical activity volume. This interpretation is in line with recent systematic reviews showing that depressive symptoms are particularly strongly associated with the mental dimension of quality of life among university students (28, 31). Importantly, the strong association observed between depressive symptoms and the mental component of HRQoL may partly reflect conceptual overlap between constructs, as both measures capture closely related aspects of psychological wellbeing. Therefore, these findings should be interpreted with caution and understood as relationships between related constructs rather than fully independent associations.

The observed association with the physical component suggests that depressive symptomatology may be associated with differences in perceptions of energy, vitality, and functional capacity, reinforcing the bidirectional relationship between physical and mental health (32). From an explanatory perspective, core symptoms of depression, such as anhedonia, reduced energy, and cognitive fatigue, may influence individuals' perceptions of physical functioning and overall self-rated health. In this regard, previous evidence indicates that depression is consistently associated with poorer perceived physical functioning and lower health-related quality of life, even after accounting for other relevant factors (33).

Conversely, physical activity was associated only with the mental dimension of HRQoL and did not show a significant association with the physical component in the adjusted model (*p* = 0.893). Although statistically significant in the model for the mental component, the magnitude of this association was small compared with that observed for depressive symptoms and should be interpreted with caution. This finding suggests that, in a young and highly active sample, perceived physical health may exhibit limited variability and therefore reduced sensitivity to differences in physical activity volume. The high levels of physical activity observed in this sample likely reflect the curriculum-driven and practice-based nature of physical education training, which may not be directly comparable to physical activity levels reported in general university populations and may also be influenced by self-report bias (34). A potential ceiling effect in perceived physical health among physically active individuals may partially explain the absence of an association with the PCS. This pattern is consistent with recent evidence indicating that psychological factors tend to show stronger associations with HRQoL than behavioral factors in university students, particularly regarding the mental dimension of quality of life, although this interpretation should be considered within the specific context of the present sample (35, 36). The present findings extend this evidence to Chilean physical education teacher education students and distinguish between the physical and mental components of HRQoL.

These findings may indicate that, in this sample of young and physically active students, the benefits of physical activity may be expressed predominantly at the psychological level, through mechanisms such as emotional regulation and reductions in perceived stress (37–39). Physical activity may therefore be associated with improved wellbeing through psychosocial pathways, including mood regulation, stress buffering, and social interaction. Likewise, the absence of an association between physical activity and PCS suggests that, in this context, higher volumes of activity did not necessarily correspond to better perceived physical health, particularly when psychological distress is present (40).

The inclusion of academic cohort in the analyses provides a developmental perspective on student wellbeing across the academic trajectory. The correlations indicated differences in sleep quality and mental wellbeing across academic cohorts; however, the descriptive data did not show a clear monotonic pattern across cohorts. This pattern may reflect differences in academic demands and professional training experiences, consistent with the literature on academic burnout and university-related stress (41). The consistency between the bivariate analyses and the multivariable model, in which academic cohort remained significantly associated with the MCS, supports the presence of cohort-related differences in mental wellbeing, although the cross-sectional design does not allow conclusions regarding changes across the academic trajectory. University environments represent complex psychosocial contexts in which academic demands, performance expectations, and career uncertainty may influence students' mental wellbeing. This interpretation aligns with recent evidence documenting high levels of mental health symptoms among university students during their academic training (42).

The sex differences observed in this study are consistent with findings from previous research in university populations, where women tend to report higher levels of anxiety, stress, and sleep disturbances, along with lower mental wellbeing (43, 44). Notably, these differences were concentrated in the psychological dimension of wellbeing, as no differences were observed in the physical component. This pattern reinforces the notion that sex disparities in this population are primarily reflected in emotional and mental health indicators, as documented in systematic reviews showing a higher prevalence of depressive and anxiety symptoms among female university students compared with their male counterparts (45). These patterns may also reflect broader sociocultural and psychosocial factors influencing emotional vulnerability and coping strategies among female students, which have been widely documented in global mental health research. These findings should be interpreted as descriptive patterns rather than indicative of broader or causal differences. These findings highlight the importance of incorporating a sex perspective into university health promotion strategies (46).

From an integrative perspective, the findings suggest that both behavioral and psychosocial factors are associated with HRQoL among physical education teacher education students. However, depressive symptoms showed substantially stronger associations than physical activity, particularly regarding the mental component of HRQoL. These results highlight the importance of considering mental health and lifestyle factors as complementary targets for health promotion within university settings (26).

Students in this sample reported high levels of physical activity, poor sleep quality, and symptoms of depression, anxiety, and stress as measured by the DASS-21. This pattern suggests that high levels of physical activity alone were not consistently associated with optimal overall wellbeing in this sample. In this regard, the findings indicate that high levels of physical activity do not necessarily coincide with optimal overall wellbeing when psychological and emotional dimensions are considered simultaneously (9). Rather than contradicting the relevance of physical activity, this finding underscores that, in this population, physical activity alone was not consistently associated with all dimensions of HRQoL assessed in the study.

Taken together, these findings suggest that approaches to student wellbeing may benefit from attention not only to active lifestyles but also to systematic psychological support strategies (47). These findings reinforce the importance of integrated health promotion models within universities that simultaneously address physical activity, sleep hygiene, and psychological wellbeing as interconnected dimensions of student health. In this sense, institutional strategies combining physical activity programs with early screening of depressive symptoms and psychological support may be relevant for supporting HRQoL among teacher education students (6). Such strategies may be particularly relevant in teacher education programs, where students are expected to become future promoters of healthy lifestyles in school contexts. Supporting their well-being during training may therefore have long-term implications for both educational environments and population health.

### 5.1 Limitations

This study hasseveral limitations that should be considered. First, the cross-sectional design prevents establishing causal relationships between the variables analyzed, limiting the interpretation of directionality in the observed associations. Second, the use of self-report instruments may introduce social desirability bias, particularly in a sample of physical education students, who may overestimate their levels of physical activity. This potential overestimation bias may have reduced variability in the physical activity measure and may have affected the magnitude of the observed associations. Third, the sample was drawn from a single university institution through non-probabilistic convenience sampling, which limits the generalizability of the findings to other academic or cultural contexts. Accordingly, the results should be interpreted as specific to this educational context and should not be assumed to be representative of all physical education teacher education students. Although the sample size was considered adequate for the number of predictors included in the regression models, it may have limited the inclusion of additional relevant confounders, potentially resulting in residual confounding. Important contextual and lifestyle factors such as socioeconomic status, living arrangements, health conditions, and academic workload were not included in the present study and should be considered in future research. Furthermore, the strong correlation observed between depressive symptoms and the mental component of the SF-12 may reflect conceptual proximity between these constructs, which should be considered when interpreting the magnitude of the observed coefficients. Finally, the high levels of physical activity reported in the sample may have generated a range restriction effect, potentially reducing the ability to detect associations with the physical component of quality of life. Furthermore, the substantially lower explained variance observed for the physical component of HRQoL (6%) compared with the mental component (61%) suggests that relevant factors associated with perceived physical health were not captured in the present study, and that the model should be interpreted with particular caution for that outcome. Future longitudinal and multicenter studies with larger and more diverse samples, and incorporating objective measures of physical activity such as accelerometry, may help to further clarify these relationships and explore potential mediating mechanisms.

## 6 Conclusion

The findings indicate that, in this sample of physical education teacher education students, health-related quality of life (HRQoL) showed stronger associations with mental health indicators than with the physical activity measure included in the models, with depressive symptoms showing the most consistent associations with both the physical and mental components of HRQoL. These findings should be interpreted within the context of a young and physically active sample, in which variability in physical activity and perceived physical health may have been limited. The differential magnitude of the observed effects, together with the substantially greater explained variance in the mental component (61%) compared with the physical component (6%), suggests that the variables examined were more closely related to the mental than the physical dimension of HRQoL in this population.

Physical activity showed a specific association with the mental dimension of HRQoL, suggesting that its association in this population was primarily associated with the psychological dimension. However, the absence of a significant association between physical activity and the physical component in the adjusted model indicates that, among young and highly active students, perceived physical health may be relatively stable and less closely associated with variations in physical activity volume. In addition, academic cohort was significantly associated with mental HRQoL in the adjusted model.

Overall, these findings suggest that approaches focused exclusively on physical activity may not fully capture the range of factors associated with HRQoL in this population. Future research should further examine the potential contribution of psychological and behavioral factors to HRQoL among university students. At the same time, the results should not be interpreted as diminishing the relevance of physical activity, but rather as indicating that psychological symptoms showed stronger cross-sectional associations with HRQoL in this specific educational context.

Given the cross-sectional design and single-university sample, these findings should be interpreted as associations rather than evidence of causal or longitudinal relationships. Future longitudinal and multicenter studies are needed to confirm these findings and to evaluate potential strategies aimed at supporting student well-being.

## Data Availability

The raw data supporting the conclusions of this article will be made available by the authors, without undue reservation.
